# Supplementary figures and images for: DNMT family induces down-regulation of NDRG1 via DNA methylation and clinicopathological significance in gastric cancer
Source: PeerJ. 2021 Sep 16;9:e12146. doi: 10.7717/peerj.12146 (PMC8450010; doi:10.7717/peerj.12146)

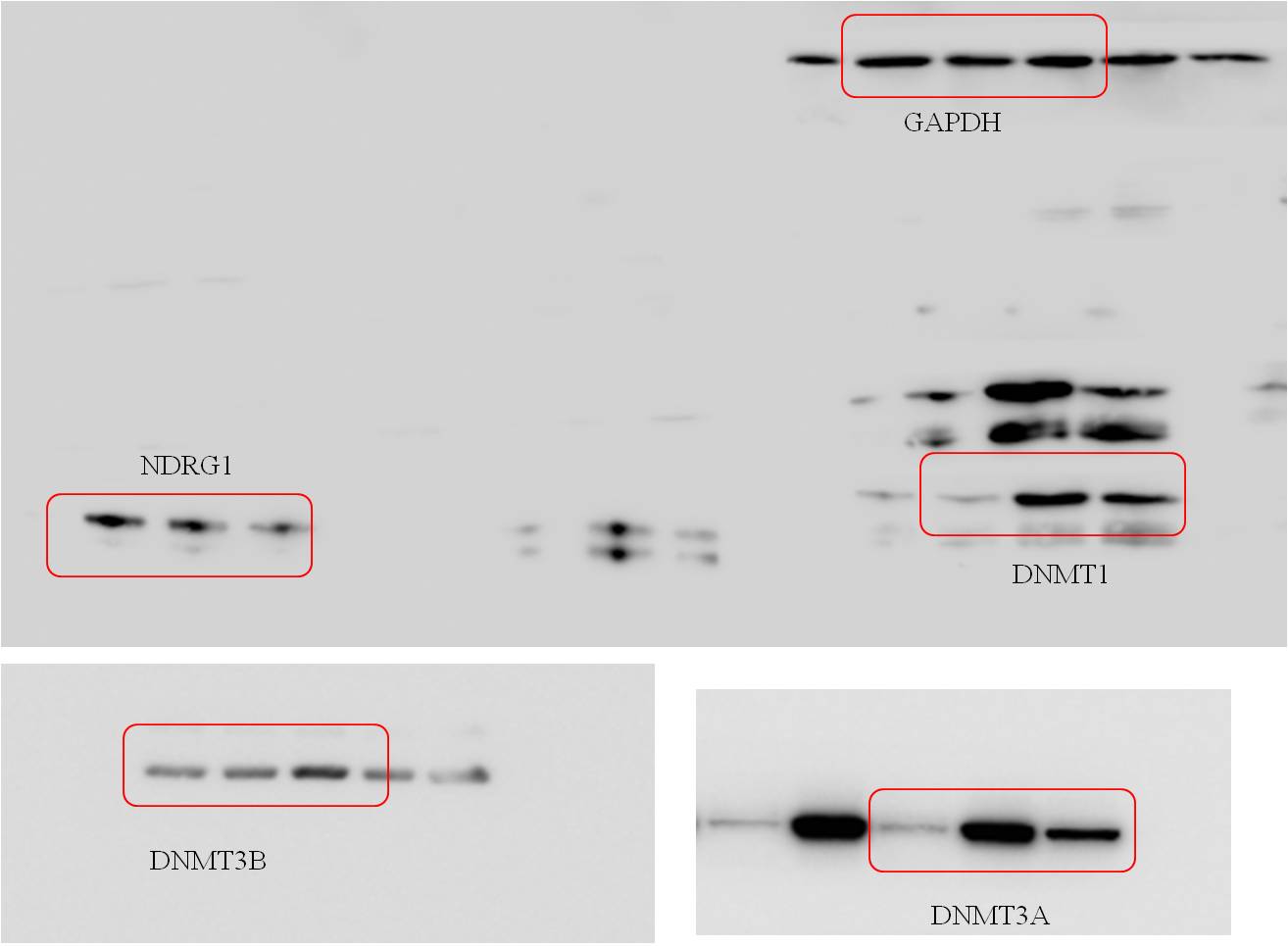

Supplement: Supplemental Information 3 [file peerj-09-12146-s003.zip › raw data-WB/WB-raw data.jpg]

Type 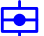 normal 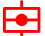 tumor

$p=0.00021$

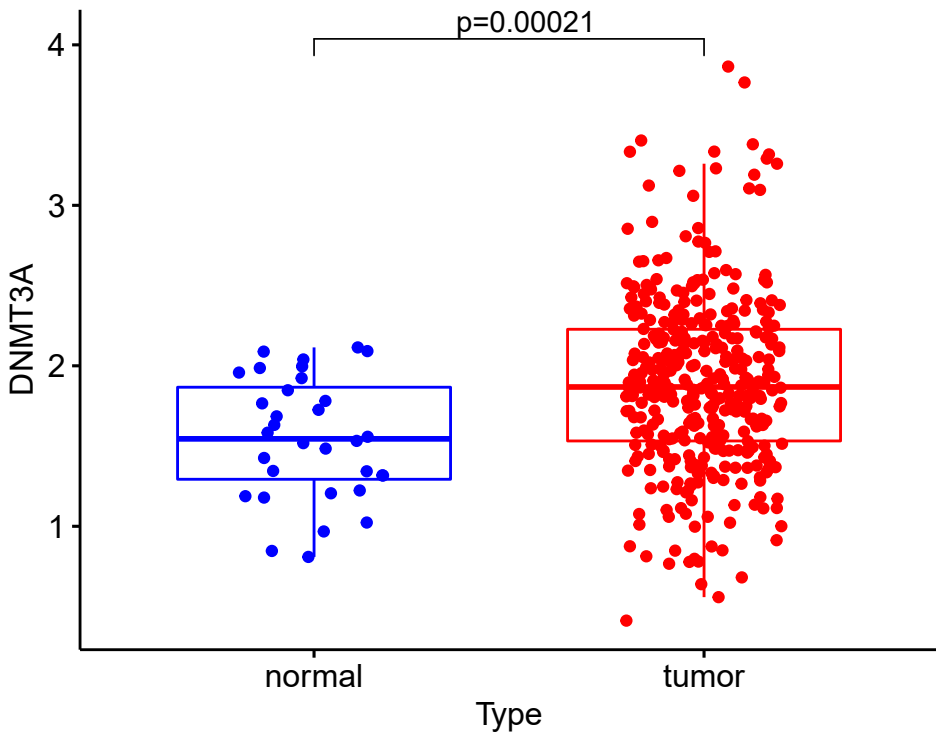

Supplement: Supplemental Information 5 [file peerj-09-12146-s005.zip › Raw data/DMNT3A-mRNA level.pdf]

Type 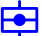 normal 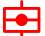 tumor

$p < 2.22\text{e-}16$

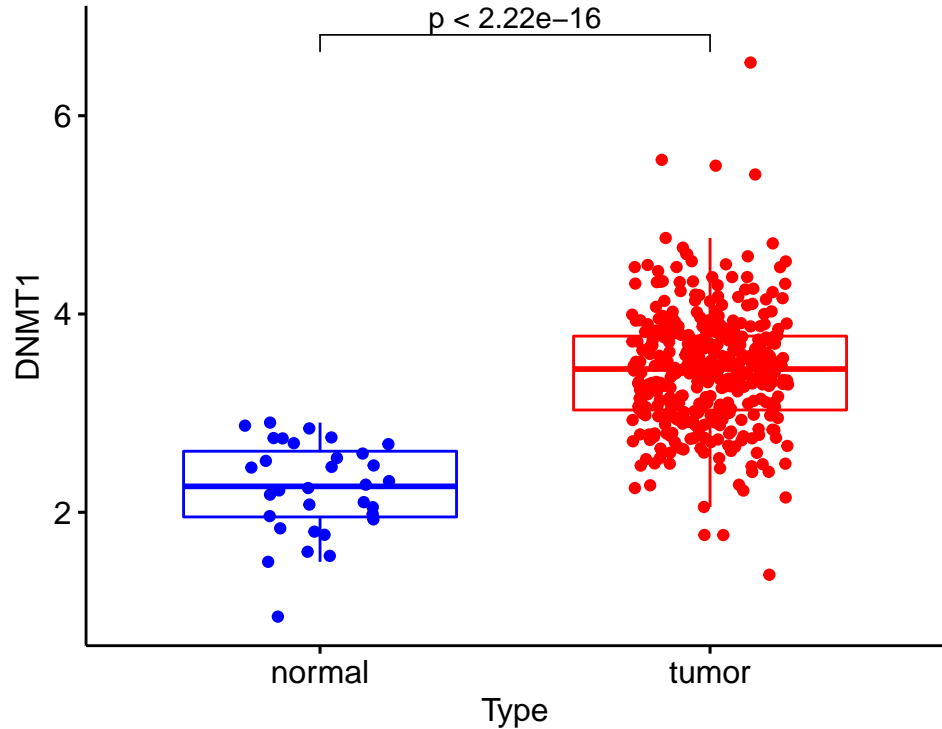

Supplement: Supplemental Information 5 [file peerj-09-12146-s005.zip › Raw data/DNMT1--mRNA level.pdf]

Type 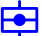 normal 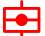 tumor

$p < 2.22\text{e-}16$

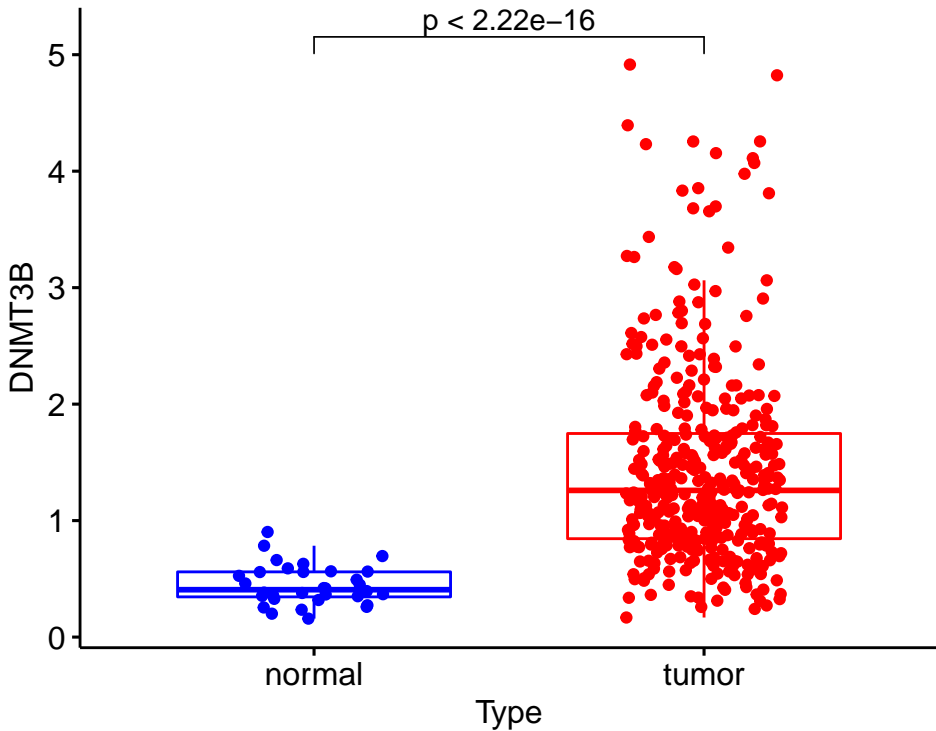

Supplement: Supplemental Information 5 [file peerj-09-12146-s005.zip › Raw data/DNMT3B-mRNA level.pdf]

# DNRG1-M-DNMT1

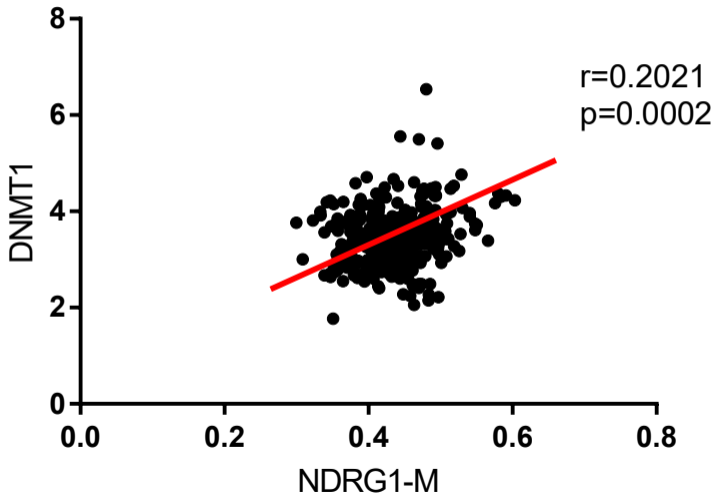

Supplement: Supplemental Information 5 [file peerj-09-12146-s005.zip › Raw data/DNRG1-M-DNMT1 (1).pdf]

# DNRG1-M-DNMT3A

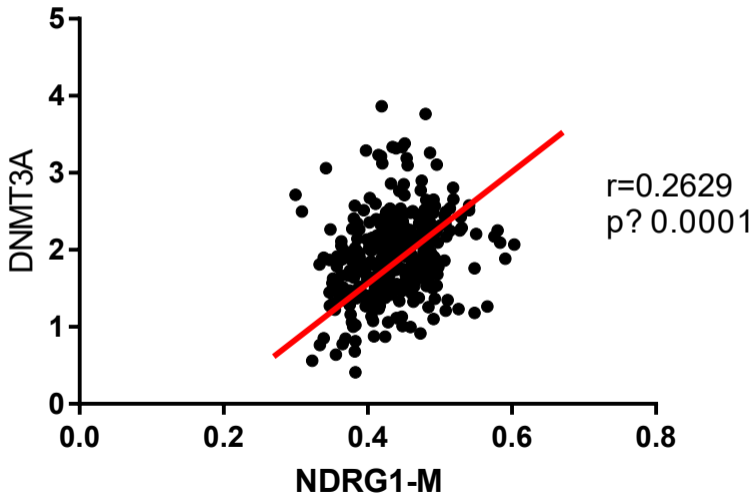

Supplement: Supplemental Information 5 [file peerj-09-12146-s005.zip › Raw data/DNRG1-M-DNMT3A.pdf]

# DNRG1-M-DNMT3B

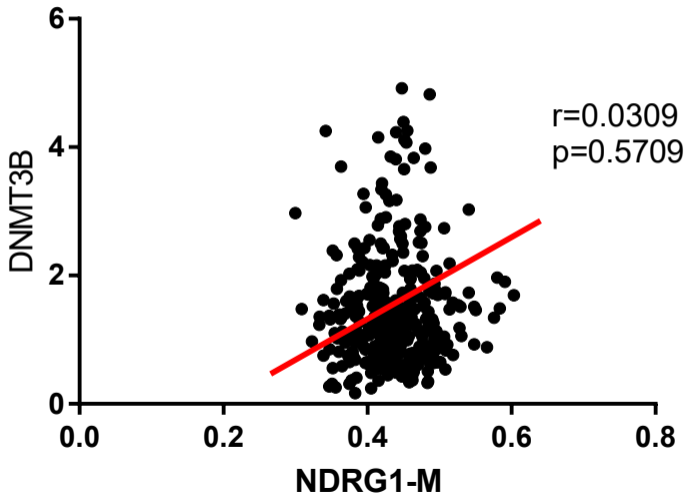

Supplement: Supplemental Information 5 [file peerj-09-12146-s005.zip › Raw data/DNRG1-M-DNMT3B.pdf]

# NDRG1-DNMT1

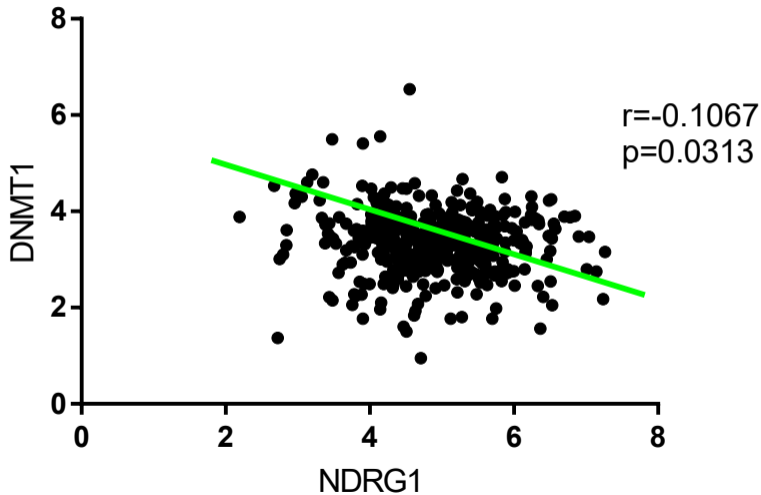

Supplement: Supplemental Information 5 [file peerj-09-12146-s005.zip › Raw data/NDRG1-DNMT1.pdf]

# NDRG1-DNMT3A

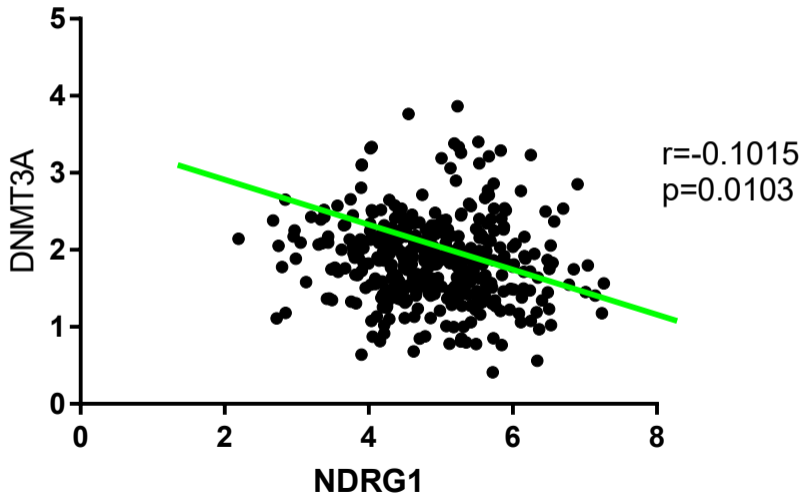

Supplement: Supplemental Information 5 [file peerj-09-12146-s005.zip › Raw data/NDRG1-DNMT3A.pdf]

# NDRG1-DNMT3B

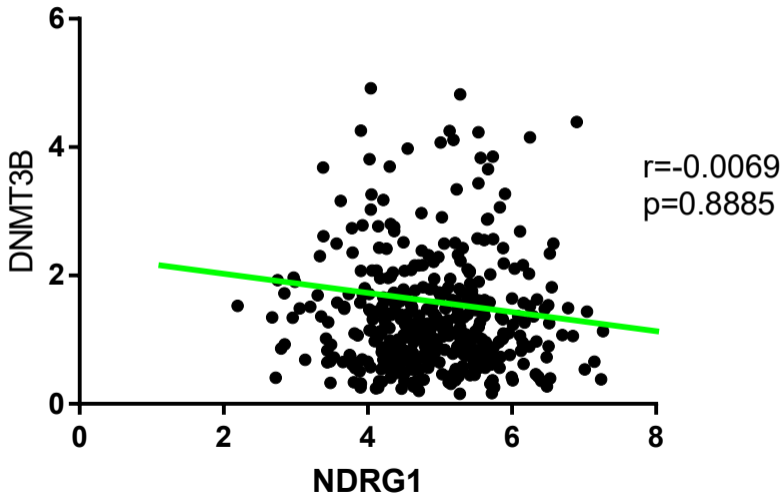

Supplement: Supplemental Information 5 [file peerj-09-12146-s005.zip › Raw data/NDRG1-DNMT3B.pdf]

Type 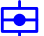 normal 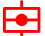 tumor

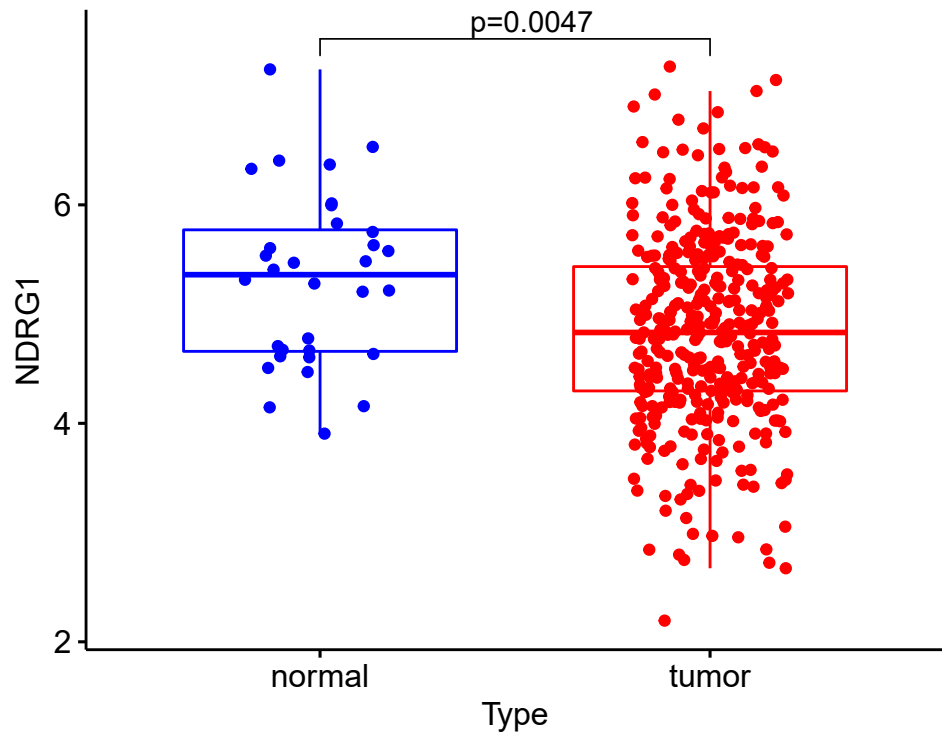

Supplement: Supplemental Information 5 [file peerj-09-12146-s005.zip › Raw data/NDRG1-mRNA level.pdf]
